# Transforming Ethiopian higher education institutions: Multilevel analysis of high-performance work systems, engagement, and justice

**DOI:** 10.1016/j.heliyon.2024.e32942

**Published:** 2024-06-17

**Authors:** Sheref Gogsido, Demis Getahun, Zerihun Alemu

**Affiliations:** University of Gondar, Gondar City, 6200, Ethiopia

**Keywords:** SHRMP, Managerial-rated HPWS, PS-HPWS, Procedural justice, Engagement

## Abstract

Perceived HPWSs (PS-HPWSs) are increasingly indicated for understanding the relationship between organizational HPWSs and employee-level outcomes. However, the mediating effect of perceived HPWSs is inconsistent and unclear. Hence, relying on integrated HPWS strength and signal theories, we tested whether PS-HPWSs positively mediated the relationship between MR-HPWSs and engagement with data collected from multiple and multilevel sources of 102 department heads and 360 employees of three Ethiopian public higher education institutions from June 03/2023 to September 10/2023. We also tested the positive moderation impact of procedural justice between MR-HPWSs and PS-HPWSs and its conditional effect on engagement using the MLmed Beta macro SPSS package. Therefore, procedural justice moderates the mediation of PS-HPWSs between manager-rated HPWSs and employee engagement. Hence, this study will address the inconsistency of PS-HPWSs between manager-rated HPWSs through the moderation of procedural justice (one of nine features of HPWS strength); this in turn has an ample effect on engagement. Accordingly, further research should include one or more of the nine HPWS features as moderators of the mediating effect of PS-HPWSs.

## 1 Introduction

Strategic human resource management practices (SHRMPs) scholars have been trying to validate the positive link between SHRMP and performance outcomes for decades, but exact theorization is lacking [1], which shows the need to verify the impact of SHRMP on organizational performance [2]. Although SHRMP researchers have traditionally focused on the nexus of SHRMP and performance outcomes at the organizational level, many scholars [[3], [4], [5], [6], [7], [8]] agree that the impact of organizational SHRMP or high-performance work systems (HPWSs) on other organizational performance can be achieved by focusing on HR outcomes/employee outcomes [9,10]. Most reported employee outcomes/employee reactions include employee engagement, organizational commitment, organizational identification, intention to leave, job satisfaction, perceived organizational support, self-efficacy, and coping with change [11]. Among them, engagement is the most cited HRM agenda as a sustainable competitive advantage variable [[12], [13], [14], [15], [16]]. Engaged employees are a source of organizational effectiveness, creativity, innovation, employee satisfaction, commitment and well-being [17]^,^ whereas disengagement is related to a higher turnover level, more safety incidents, and less profitability [18]. Therefore, to have both organizational and employee mutual benefits, HPWS must first affect lower-level variables such as individual-level attitudes and behaviors [6,[19], [20], [21]]. In line with this, many researchers have shown that organizational HPWSs have a positive impact on employees’ attitudes, including engagement [14,22]. However, little is known about the effectiveness of human resource management in the public sector [[23], [24], [25]], although there is no difference between the public and private sectors in terms of the impact of human resource management on intermediate outcomes (engagement) [26]. According to a 50-year meta-analysis, studying the impact of HRM on engagement in the public sector is crucial, particularly in HEIs [27].

However, what human resource management practices or systems are most important for employee engagement and the theoretical mechanisms that intervene in and explain the link between HPWSs and employee engagement are not clear [13,17,28]. Four conceptual models have proposed that HR perception is a crucial mediator in the relationship between MR-HPWS and employee attitudes and behaviors [19,[29], [30], [31], [32], [33], [34]]. This shows that scholars may not refer to HR perception^1^ in the same dimensions, so an increasing number of studies have perplexed HR perceptions with other related conceptualizations [35]. Although many studies have investigated the role of perceived organizational support, including perceptions of fairness, job conditions, supervisors and rewards, organizational justice, leadership and manager integrity equity, service climate, leader member exchange, and transformational leadership [36], only a few studies have recently investigated the role of employees' perceptions of HPWSs [37] as a mediator between MR-HPWSs and attitudes. Engagement is an activating job attitude, as shown to be related to PS-HPWS [38]^,^ so employees’ view of HPWSs seems to be more appropriate than the managerial-rated view of HPWSs [39]. It is claimed to perform better than traditional only managerial-rated HPWS in terms of productivity, favorable HR climate, flexibility, return on investment in human resource management, and firm financial performance, so it is a better source of sustainable competitive advantage and an extra means of organizational effectiveness [40]. While its main focus is mediating the relation between MR-HPWSs and engagement [32,35,41]^,^ there is a conflict of knowledge about the conceptualization and components of HPWSs [31,35,[42], [43], [44]]. Similarly, even PS-HPWS mediation is not consistent because there are three conceptualizations and factor structures: PS-HPWS content, the HPWS process and HPWS attribution [35,[45], [46], [47]]. To answer this question, there is a need for a comprehensive conceptual model of PS-HPWS as a mediator between MR-HPWSs and engagement through the intermixing of HPWS content and HPWS process views (HPWS strength), which responds to a call for studies [48,49].

Primarily, when we refer to PS-HPWS, it is the HPWS content [35], not strength. However, researchers have observed underestimation in evaluating the essence of PS-HPWSs [35] as well as their determinants [35,37,[50], [51], [52], [53]]. Consequently, a small number of empirical studies [[54], [55], [56]] have agreed that PS-HPWSs are a mediator between MR-HPWSs and employee outcomes [35,57], particularly with regard to engagement [32] in public HEIs in Ethiopia. In contrast, many prior studies have argued that even if employees' collective perceptions of HPWSs are important, they are inconsistent with managerial-rated HPWSs [29,32,37,54,58,59] in affecting employees' outcomes according to signal theory [48,[60], [61], [62]]. For example, empirical evidence from many scholars shows that the average correlation between manager and employee perceptions of HPWSs is moderate (*r=*0.37 on average) [35] and weak [29,63] and that in turn has a low impact on employees' outcomes [31,54,55,64]. Consequently, the possible inconsistency between HPWSs rated by managers and those perceived by employees is welcomed by scholars, and scholars need to focus on the study of PS-HPWSs’ mediation between managerial ratings and HR outcomes [35,57], such as engagement.

First, currently, low-level engagement is due to inconsistency between MR-HPWSs and PS-HPWSs, which can be minimized through the fit concept of dual aspects of HPWSs (commitment and control-oriented HPWSs) based on signal theory [65]. Likewise, there is a call for studies to employ different measurements for the existing multilevel homologous HPWS construct [11], focusing on the context-driven measurement scales of PS-HPWSs [66]. The contextualization of human resource management is related to the consideration of external and internal environments within HPWSs [67]. This highlights the need for both external consistency between HR practices and organizational strategy and internal consistency among HR practices [68]. In other words, consistent human resource management systems need commitment- and control-oriented HPWSs to impact employees' attitudinal outcomes [47,65,69]. However, the understanding of HPWSs is limited and incomplete and misplaces the value of control-oriented HPWSs because only a few HPWS studies have considered both commitment- and control-oriented HPWSs simultaneously in exploring how the dual interpretation of HPWSs affects employees' engagement [70,71]. Signal theory has proposed that signal fit to both MR-HPWSs and PS-HPWSs affects employees’ attitudes [35,49,60,61], including their engagement. Hence, the PS-HPWS meditational hypothesis is aligned with signaling theory [60,72], which shows that strong dual managerial-rated HPWSs produce signals about what is expected and valued, which are reflected in reports of perceived dual HPWSs and subsequently influence employee outcomes [11,73,74]. Hence, we employed signal theory to mediate strong perceived integrated dual HPWSs between managerial-rated integrated dual HPWSs and engagement.

Second, as we discussed above, a number of scholars have argued that PS-HPWSs tend to be lower than manager-reported HPWSs [32,37,55,58] due to the weak effect of managers on PS-HPWSs at the collective level, which in turn decreases employee outcomes [19,42,46,75] such as engagement. This shows that an insignificant number of studies have been carried out on what factors could strengthen the alignment between MR-HPWSs and PS-HPWSs [37,57] in impacting employees' outcomes [[31], [32], [33],[54], [55], [56],^58^]. Previous studies have investigated the moderating variables rated by managers, including employee-oriented organizational culture and CEOs' benevolent leadership behavior [76], managers' communication quality [55], leader-member exchange and trust [77]^,^ between organizational HPWSs and PS-HPWSs and their interaction with employees’ attitudes. Nevertheless, there are calls for research to study the strengthening factor/s of (mis)alignment between MR-HPWSs and PS-HPWSs [29,31,32,59]. The main proposed moderating factor includes nine meta features of HPWS [48,49], particularly consensus (one of three main features of HPWS strength) [35,75]. Hence, based on signal theory, there is an imbalance between MR-HPWSs and PS-HPWSs [29,31,32,59,60] that can be moderated through nine meta-features of HPWSs [48,49], particularly consensus (one of three main features of HPWS strength) [35,75]; consequently, the engagement of employees decreases [78]. Accordingly, based on signal theory complemented by HPWS strength features, Guest et al. [48] showed that agreement among HPWS implementers (one of the consensus features or one of nine meta-features of HPWS strength) moderates the impact of MR-HPWSs on PS-HPWSs. Here, we conceptualized the other aspect of consensus (justice of fairness of HPWS mainly with performance management) [79]. Since justice provides employees with expectations of the messages sent by line managers [11,73,80,81], it should be perceived by employees. Regarding the type of justice, Biswas et al. [82] concluded that both procedural and distributive justice perceptions need to focus on evaluating support from employers. The similarity between procedural and distributive justice becomes more apparent since both justices are derived from individuals' expectations about outcomes [83]. Therefore, we assumed that perceived procedural justices (dual dimensions) positively moderate the mediation of PS-HPWSs between MR-HPWSs and engagement at the department level.

We were motivated to carry out this empirical study in Ethiopian public HEIs for the following reasons. **First**, Ethiopia is a country with a large economy; currently, it has the largest economy in East Africa (US$111.3 billion in 2021), and it is the fourth largest economy in sub-Saharan Africa [84]. Similarly, strong improvements in the social well-being of Ethiopians also reflect the country's development progress, as between 2000 and 2021, Ethiopia's human development index value increased by 73.5 % [84]. Economic and well-being development is one of the motives for study in Ethiopia. **Second**, although there is growing recognition of public-driven role changes to private sector and foreign investment roles, Ethiopia is well known as a public sector-driven development strategy for every sector of the country [85]. That is why the study is in the public sector. **Third**, in Ethiopia, improving the quality of public HEIs is crucial for improving overall public sector services by providing qualified graduates to the public service and governance system [86]. Since HEIs are strategic focuses of the country, we are beginning to carry out HEIs. **Fourth**, while technological, economic, social and demographic paradoxes are occurring, institutional approaches (new national administration philosophy) to universities present new insights into the logics of change and maintenance under these increasingly difficult conditions [87]. Hence, globalized patterns of excellence and reforms have truly transformed organizations and relations in the higher education and research sector [88,89]. The emergence of HPWSs was in part a response to competitive forces in the increasingly global environment [68]. This shows that changes in national management philosophies need to be evaluated from the SHRM perspective via employees' engagement and perceptions of their managerial practices.

**Fig. 1:**
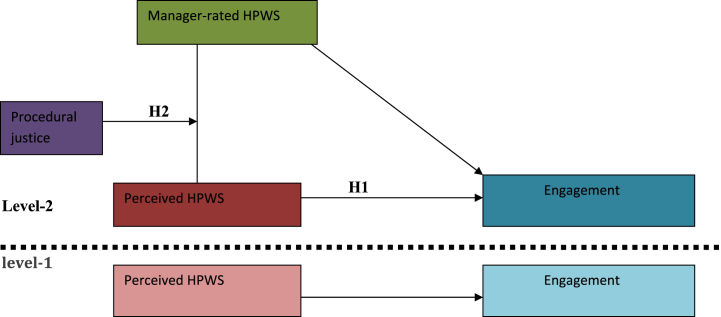
Researchers own model that shows moderation of procedural justice on the meditation impact of PS-HPWS between manager-rated HPWS and engagement.

This study has theoretical, methodological and practical value. This study uniquely emphasizes three **theoretical values** for SHRMP scholars. First, we argue that in signal theory [60]^,^ to increase employee engagement, employees must first experience HPWSs, which are the same as MR-HPWSs [60,78]. This increases its application in the mediation of PS-HPWSs between MR-HPWSs and attitudes (including engagement) [60,61,78]. Second, based on the recommendation of PS-HPWS meditation hypothesis development by signaling theory [60,72], strongly integrated dual (fit relative to unfit of dual) MR-HPWSs invariably affect the perceived fit of integrated dual signals (HPWSs) by employees and subsequently influence employee outcomes [49], specifically engagement [78]. Hence, we extended SHRMP theory by introducing fit of signal (HPWS) in applying PS-HPWS [49] mediation between MR-HPWSs and engagement because the fit of HPWS decreases the inconsistency between MR-HPWSs and PS-HPWSs, which in turn enhances the engagement of employees. Previously, homologous HPWSs rated by managers and perceived by employees and their impact on engagement were not dually dimensioned. Third, to enhance the work attitude of employees, the impact of MR-HPWSs on PS-HPWSs needs to be strengthened with nine HPWS strength features [48,74], particularly by consensus [48] (procedural justice, see Fig. 1). This study addresses the current inconsistency in perceived HPWS mediation between MR-HPWSs and engagement. Side by side, we extended via the integration of HPWS content and the HPWS process (HPWS strength theory), which responds to a call for studies [48,49].

**Methodologically**, it extends the application of multilevel mediation and multilevel moderation analysis under a multilevel structural equation model, which is repetitively stated by SHRMP researchers [[90], [91], [92]]. **Practically**, as we discussed, engagement is a source of strategic competitive advantage for institutions, but it is not simply possible to engage employees in their performance. Hence, managers could determine how to enhance the engagement of employees in their role because this study suggests that the perception of HPWSs with fit could increase engagement better than manager-rated HPWSs. Again, procedural justice is used to strengthen the impact of department heads on the perception of HPWS, which in turn impacts the engagement of employees.

## 2 Literature review

### 2.1 Meditating effect of PS-HPWSs on the relationship between MR-HPWSs and engagement

SHRMP studies have substantially improved the understanding of the relationship between HPWSs and employee outcomes (including employee engagement, organizational commitment, organizational identification, intention to leave, job satisfaction, perceived organizational support, self-efficacy, and coping with change) [65,93]. Engagement is more strategic than others in terms of both employees and organizational outcomes [14]. Even if research on employee engagement has demonstrated that HPWSs are positively related to employee engagement [13,94,95], it is also not clear what HPWSs are most important for employee engagement and what theoretical mechanisms intervene in explaining the link between HPWSs and employee engagement [13]. Hence, more explanation about the relationship between HPWSs and engagement, particularly focusing on mediation, is needed. The explanations are directly or indirectly related to the following points.

First, rather than organizational-level HPWSs, MR-HPWSs should be focused on enhancing engagement [17] because line managers are sense makers who send HPWSs to employees according to signal theory [31]. By ‘locally modifying HPWS, line managers can shape the horizontal and vertical fit of these practices to improve employees outcome’ [96,97]. As direct managers of operational employees, line managers are sense makers to employees through HPWS [46]. However, a few studies have focused on HPWSs at the team level [31]. Hence, studies that focus on manager-rated HPWSs are essential for enhancing employee outcomes [98].

Second, to further enhance engagement, line managers' views of HPWSs and employees’ perceptions of the same HPWS were unplugged by scholars [17,99]. Since PS-HPWS by employees influences employee behavior more than MR-HPWS does [35,36,53], many scholars are increasingly studying the relationship between MR-HPWSs and outcomes [35,48,57,78,100]. Empirically, the positive impact of MR-HPWSs on PS-HPWSs is well documented in the literature [11,35,37,57]^,^ as is the positive effect of PS-HPWSs on engagement [3,18,35,101,102]. These studies support the mediation of PS-HPWSs between MR-HPWSs and attitudes (e.g., engagement) 36,94). However, there are only a few studies in this area [31,55,64] that show the need for additional empirical tests. Of the range of social exchange theories [103], signal theory [104] is one of the most broadly employed theories in studies conducted at the micro level exploring the line manager-employee relationship and its effect on employee attitudes and behaviors [98]. Although the majority of human resource management and organizational behavior studies have examined signaling in talent attraction and recruitment processes, few studies have investigated information asymmetry between MR-HPWSs and PS-HPWSs and its effect on employee attitudes [61]. In line with recent recommendations of signal theory to study such relationships and interventions [11,48,60,74,78], we applied signals as an overarching theory to study the mediation of PS-HPWSs between MR-HPWSs and engagement.

Third, the main mediating variable between MR-HPWSs and employee attitudes and behaviors is employee perception of HPWSs [19,29]; however, the most challenging concept is still how individual-level perceptions of HPWSs can emerge at a higher level. This shows that the knowledge gap about how managerial-rated HPWSs impact engagement could be useful at the organizational level [20]. To solve this problem, Ostroff and Bowen [19] distinguished coherent management values to influence individual ‘psychological and collective’ employee perceptions of HPWS or team climate and their impact on individual- and team-level outcomes, respectively [30,105,106]. Signal theory also proposes such differentials (see 72). Hence, lower-level perceptions of the impact of HPWSs on engagement become aggregated to form upper-level collective perceptions of the impact of HPWSs on engagement [19,29]. However, there is an inconsistency in the meditational effect of PS-HPWSs between MR-HPWSs and engagement because one group of scholars states that there is parallel mediation of PS-HPWSs at both the upper and lower levels [76], whereas the other group believes that the mediation of PS-HPWSs at the upper level still accepts the impact of individual-level PS-HPWSs on engagement at the lower level [55]. This shared employee perception of HPWS (sometimes referred to as the team climate) leads to organizational performance [29,32,54,55,77]. We considered the impact of lower-level PS-HPWSs on engagement to be consistent with the discussions of Xiao and Cooke [66] and Beijer et al. [107] and considered only the department-level mediation of PS-HPWSs between MR-HPWSs and engagement collectively or aggregately. Previous studies have shown that PS-HPWSs positively mediate MR-HPWSs and employee outcome [engagement] [3,77] only at the upper level [31,32]. However, only a small number of such studies [35] have focused on disaggregating within-group and between-group terms [90], particularly in the public HEIs of developing countries.

Fourth, according to signal theory, the argument of Meier-barthold and Alfes [65] shows that HPWSs lead to more engagement among employees when control- and commitment-oriented HPWSs are balanced rather than unbalanced. The perceptions of both commitment- and control-oriented HPWSs mediate managerial-rated integrated dual HPWSs and engagement [108]^,^ where control-oriented HPWSs include performance appraisals and performance-based pay, while commitment-oriented HPWSs are related to intensive recruitment and selection, extensive training and development, participation, information sharing and others. Accordingly, duality is defined as a control-oriented HPWS (such as results-oriented appraisals and profit sharing) that exclusively comprises practices directed toward efficiency and competition and therefore signals productivity, whereas a commitment-oriented HPWS (such as internal career opportunities, extensive training and development, employment security and participation) that focuses on long-term employment relationships within the organization signals commitment [65]. Relying on the work of Alfes et al. [102], we conceptualized the perception of integrated dual aspects of HPWSs as positively mediating managerial-rated interactive dual HPWSs and employee engagement.

Hypothesis 1 PS-HPWSs positively mediate the relationship between MR-HPWSs and engagement at the department level.

### 2.2 Moderation effect of procedural justice on the meditational effect of PS-HPWSs between MR-HPWSs and engagement

According to signal theory, there is usual asymmetry between MR-HPWSs and PS-HPWSs and their impact on engagement because of idiosyncratic deals in the perception of HPWSs (that is, employees rely on their experience when there is less understanding of HPWSs, e.g., 46,52,72). Such inconsistency shows a need for moderating variables [57]. Signal theory^2^ “does not only pay attention to the quality of the signal itself by tapping into the characteristics of clarity, observability, frequency, and consistency”, but it also indicates other factors “that are likely to be important for HR-related communication such as the characteristics of information senders and receivers, and environmental distortion of messages” [11]. This implies that signal theory proposes not only the mediation of PS-HPWS but also the moderated mediating effect of the characteristics of line managers and employees as well as environmental distortion. Obliviously, the moderating variables studied in PS-HPWS studies are not among these three main areas of moderating factors. Researchers have proposed moderating factors [51,66], including demographics (e.g., age, gender, education, socioeconomic background), intrapersonal characteristics (e.g., beliefs, values, and personality traits), family dynamics (e.g., parental relationships) [93,109,110], and cultural values (e.g., country-level power distance) [52], between MR-HPWSs and PS-HPWSs and their conditional effects on engagement. Accordingly, the empirical evidence on the moderating variables between MR-HPWSs and PS-HPWSs includes manager communication quality [55], demographic similarity with managers [37,111], employees' social status [31], employee-supervisor relationships [112], perceived employee-oriented organizational culture and top managers' benevolent leadership behavior (but in relation to firm HPWS [58]), cultural value similarity and employees’ cognitive cultural intelligence [57].

However, recently, scholars have proposed the use of HPWS strength to fill the gap between MR-HPWSs and PS-HPWSs and their effect on engagement, but this strength is dispersed [46]. This shows the need for more empirical work. This low level of employee engagement is related to the inconsistency of managerial-rated HPWSs and PS-HPWSs according to signal theory [45,113] and can be moderated through HPWS strength features [46,48,74]. How the idiosyncratic perception of HPWSs predicts PS-HPWSs at the employee level when HPWS strength is weak, which is due to ambiguous situations, is interpreted based on direct experience; in contrast, a strong situation reduces the dependence on the individual experiences that make perceptions of PS-HPWS climates more homogenous with one another [114]. In other words, HPWS strength makes the impact of MR-HPWSs on PS-HPWSs more collective and then impacts the collective outcome of employees; otherwise, idiosyncratic perceptions and their causal relationships will be formed at a lower level. However, of the three features of HPWS strength, consensus is the focus of most related studies. While scholars have proposed the importance of obtaining consensus with regard to organizational HPWS in promoting collective outcomes [75], much less is known about ‘the organizational factors that promote strengthen the relationship between upper-level HPWS and PS-HPWS at aggregate level’ [48,58,74]. This means that an upper-level construct (it may be an aggregate) of consensus is needed. The consensus includes both managers' agreement about HPWS implementation and perceived justice [45,115]. Previously, the consensus of manager agreement on HPWS implementation was studied as a moderating variable for MR-HPWSs and PS-HPWSSs and their effect on employee outcomes [48,114]. However, there is a need for a climate of perceived consensus or justice [115] to promote the integration of interests between managers' and employees' perceptions and their consecutive effects on employees' outcomes [116]. Consequently, this implies that the signal theory assumption of frequent inconsistency between MR-HPWSs and PS-HPWSs and their effect on employees' outcomes can be minimized through the perception of a justice climate (one of three main features of HPWS strength).

Similarly, Biswas et al. [82] argued that the fairness of managerial policies and practices, especially managerial-rated HPWS, forms the informational source of employees' perceptions of HPWS, which shapes their cognizance of the various groups or individuals and thus their outcomes [79,117]. This means that fairness moderates the impact of MR-HPWSs on PS-HPWSs and their consequences for engagement. However, there are different forms of justice that are intertwined with performance management [118]. Even if Colquitt et al. [118] argued that performance appraisal justice can be divided into four dimensions (namely, procedural justice, distributive justice, interpersonal justice, and informational justice), Greenberg [119] first proposed that employees' perceptions of performance appraisal justice include two dimensions: procedural justice and distributive justice [120]. Hence, we considered these two forms of justice as procedural justice within the concept of performance management. In summary, the consensus or fairness of managers that can be perceived by employees moderates (strengthens) the impact of actual managerial-HPWS implementation on employees' perceptions of the same HPWS [19] and their effect on employee outcomes. This shows the moderated mediation analysis [[121], [122], [123]] of perceived procedural justice by which those who perceive management as less fair can be expected to view many HPWSs with more disbelief than employees who view management as fair, and then, they will be disengaged [118,[124], [125], [126]]. Hence, by complementing the strength of the HR systems of Bowen and Ostroff [75] and signaling theories of Connelly et al. [60], we suggest that employees’ collective perceptions of HPWSs are more likely to emerge and align with managerial-rated HPWSs [11,58] at the collective level when perceived procedural justice is high such that the meditational impact of PS-HPWSs between managerial-rated HPWSs and employee engagement becomes high. This topic has not yet been studied.

Hypothesis 2 Procedural justice positively moderates employees’ PS-HPWS meditation effect between MR-HPWSs and employee engagement, as the mediating effect of PS-HPWSs between MR-HPWSs and engagement increases when procedural justice is stronger rather than weaker.

## 3 Materials and methods

### 3.1 Study location

In Ethiopia, there are 47 public higher education institutions, including polytechnic universities, which have recently been categorized into four categories. This includes 8 research universities, 15 applied universities, 21 comprehensive universities, and 3 specialized universities. Researchers selected one from applied universities (Woliata Sodo) and two from comprehensive categories (Wachemo and Worabe). In terms of location, Woliata Sodo University is located in Sodo town (the political and administrative city of southern Ethiopia's regional state and capital of the Woliata zone). Wachemo University is located in Hossana town (a political and administrative city of the central Ethiopian regional state and capital of the Hediya zone). Worabe University is located in Worabe town (capital of the Siltie Zone). All of these universities are located in the southern part of Ethiopia.

### 3.2 Source of data collection

Researchers utilized both primary and secondary sources of data [127]. We employed a primary source of data according to signal theory [48,60], and prior researchers [31,[55], [56], [57],^128^] showed that employees (teaching staff) and line managers (department heads) are appropriate informants regarding the actual HPWS implemented by line managers. The profile of respondents (minimum work experience and inclusion of diversity) was obtained from secondary sources.

### 3.3 Sample units, sample size determination, sampling strategy and procedure

**Sampling strategy and procedures**: Since the sampling technique for the multilevel design is a multistage sampling technique [129], first, using simple random sampling, we selected three universities from 47 public universities, namely, the Wachemo, Worabe, and Woliata Sodo universities. The randomness of this step does not matter because the sample size can be selected on the next steps of a team-level study. Second, from each university, departments are selected using a stratum sampling technique based on the college, institute and school classification. From each department, five employees were selected using a simple random sampling technique. Hence, at the departmental level, each department head completed the questionnaire.

**Sample size determination:** For the multilevel analysis, Maas and Hox [130] proposed at least 50 groups, and the number of members in each group should be at least 3–5 persons. However, for the multilevel structural equation model, at least 100 department-level sample sizes are recommended. Accordingly, we considered 149 groups and five respondents from each department. In other words, 745 employees were selected. Our planned sample included 90 % of departments from three selected Ethiopian universities.

**Sample unit or inclusion criteria at both levels**: Based on the study of Kaufman, Wilkinson, Barry and Gomez [116], department heads were screened to omit heads who had less than two years of experience. On the other hand, based on previous study of Steffensen Jr et al. [131], we included employees who had more than 2 years of work experience. In line with Kaufman et al. [116], we did not include employees who had less than 2 years of experience, who were less than 18 years old, or who were contract employees. Moreover, the overall profiles of both department heads and lecturers are displayed below (see Table 1).

**Table 1:** Shows demographic profile of department heads and lecturers.

Demographic profile	Scales	Department heads		Employees lecturers	
		Figure	Percentage	Figure	Percentage
Gender	Male	86	84.31	265	73.62
	Female	16	15.69	95	26.38
Age	18–25	5	4.9	40	11.11
	26–35	61	59.80	275	76.39
	36–45	26	25.49	35	9.72,
	46-55,	6	5.82	6	1.67
	56–60	4	3.82	4	1.11
Marriage status	Married	77	75.49	242	67.22
	Unmarried	25	24.51	110	30.56
	Divorced	–	–	8	2.20
Educational status	BA.	5	4.90	21	5.83
	Masters	64	62.74	315	87.50
	Asst. prof. & masters	25	24.51	19	5.28
	Asst. prof. & Dr.	8	7.84	5	1.39
Work experience	2–5	27	26.47	204	56.67
	6–10	44	43.13	109	30.28
	11–20	21	20.59	36	10.00
	Greater than 21	10	9.8	11	3.06

**Response rate**: Questionnaire data from 109 department heads and 380 employees were collected from June 03/2023 to September 10/2023. After removing unmatched questionnaires, 102 heads' and 360 employees’ questionnaires were included in the analysis. Therefore, 102 (68.45 %) department heads and 360 (48.32 %) employees responded effectively. The overall response rate was 51.68 %, which is greater than the average suggested in the literature for multilevel studies [132]. In addition, small sample sizes at the employee level do not pose a problem by themselves (even with 1 group size is enough, but a larger sample size is better) [133]. To improve the analysis, we included at least 3 employees within one group.

### 3.4 Data collection

**Data collection tool and duration**: We collected data using a self-administered questionnaire. A questionnaire is one of the most widely used tools for collecting social science data and helps individuals obtain relevant data or test a hypothesis [134] in a reliable and valid manner [135]. In particular, self-administered questionnaires provide autonomy to respondents, ensuring a high response rate and minimal interviewer bias and increasing the degree of personal contact [136].

**Data collection procedure** First, we obtained institutional approval from academic vice-presidents and/or academic directorates of universities to validate the legitimacy of the selected universities. Hierarchically, approval was also obtained from college and/or institution heads. Then, the department heads of each university were contacted to help them understand the purpose and procedure of the survey. The employee questionnaire was first sent by the department heads, but most of the employees returned the questionnaire through physical means (by contacting our phone number) that ensured employee confidence. The department heads of many groups also endorsed me in collecting questionnaires from employees. In the questionnaire, the respondents were assured of confidentiality and that no one from the universities would have access to their individual responses. To further reduce potential psychological stress, we did not include any questions in the employee surveys pertaining to individuals’ names or departments. Since the specification of the department in the questionnaire may be a concern for employees while it is necessary in the multilevel research design, we completed departmental identifications at the collection time of the questionnaire.

### 3.5 Questionnaire development process

Three phases of tool development were carried out. In the first phase, face and content validity were assessed by experts. We followed the same procedures used in the previous management work of Koednok and Sungsanit [137]. Accordingly, we contacted 3 doctors (PhD degree holders) and assistant professors who had publications. The second phase of tool development was carried out to translate and redesign the questionnaire, including the word order and layout. The translation process was carried out by adapting the Brislin translation model [138] procedure. Because Amharic is the official language of commerce and administration in Ethiopia, we translated it to Amharic. In the third phase, we employed a pilot test using 50 employees from the Hossana polytechnic college.

### 3.6 Measurements

***Manager-rated HPWSs:*** MR-HPWSs were measured using 10 measures with 54 items. We operationalized, in line with Meier-Barthold et al. [49], the HPWS configurations by designing HPWSs, which consisted of the same 10 HPWSs, including job design, recruitment/selection, training/development, performance evaluations, compensation/rewards, promotion/career, employment security, participation, employee voice/grievance, and information sharing. We replaced employee voice with autonomy, as many researchers [14] have argued that autonomy is the best of all practices. Then, items were developed based on related literature. In total, 54 items of the MR-HPWS were rated using a five-point Likert scale. The scale's Cronbach's coefficient was 0.924.

***Perceived HPWS***: The same question with a slightly different format from MR-HPWS was employed. Similarly, the question format from Dorta-Afonso et al. [139] assesses the constructs of “employment security” (e.g., my job is guaranteed in this department), “training” (e.g., the department provides training to adapt new employees to their work), “opportunities for advancement” (e.g., the department provides me with real opportunities for promotion and advancement), and “results-oriented appraisal” (e.g., the department values my performance at work objectively). The Cronbach's α was 0.930. The ICCs of employees' perceptions (ICC1 is 0.176, ICC2 is 0.918) provided support for our aggregation of employees' individual perceptions into collective perceptions at the department level [140].

***Procedural justice****:* Justice measurement is based on procedural justice assessed with 9 items of the scale adopted Erdogan et al. [141]. Responses were rated using a five-point Likert scale ranging from 1 (strongly disagree) to 5 (strongly agree). The questions included “The process used to conduct my performance appraisal is fair”. The Cronbach's α was 0.886. The ICC1 and ICC2 values of employees' perceptions of procedural justice (ICC1 is 0.338; ICC2 is 0.832) support the aggregation of individual-level procedural justice to collective-level procedural justice/departmental-level justice.

***Employee Engagement:*** We used an employee engagement scale that consists of 12 items that are equally distributed across the three factors (emotional, behavioral, cognitive) of Kahn's [142] engagement theory and measured by a 5-point Likert scale (1 = completely disagree, 2 = disagree, 3 = neutral, 4 = agree, 5 = completely agree) [143]. The scale's Cronbach's coefficient was 0.856. We applied a direct consensus composition model for team-level engagement because, first, engagement conceptualized and operationalized at the individual level is functionally isomorphic to collective engagement at the department level. Therefore, to ensure that engagement was acceptable for aggregation at the department level based on Bliese [144], we assessed the ICCs of the levels. The ICC1 value is 0.276, and the ICC2 is 0.823.

***Control variables***. Because of the multilevel data structure, this study controlled for factors at both the individual and department levels. At the individual level, we controlled for gender, education, marital status, work experience, and income in the analyses due to their potential impacts on employees' individual perceptions of HPWS and their work outcomes [32]. At the departmental level, we controlled for departmental managers’ age, gender, education [29], marital status, and experience as control variables because these factors may influence how they perceive HPWS and implement HPWS in their departments.

### 3.7 Data analysis method

Researchers have also assessed descriptive statistics to test missing data, outliers, normality and multicollinearity [145]. Then, a correlation test was performed to evaluate the reliability of the relationships between variables. Next, confirmatory factor analysis was carried out to evaluate the validity of the findings. Finally, multilevel moderated mediation was carried out to test the hypotheses.

We analyzed the multilevel structural equation model using two software programs. The measurement model, which is a structural equation model, was evaluated using Jamovi, whereas the structural model, which is a multilevel part, was analyzed using the software package called the MLmed Beta 2 macro package in the SPSS software [146] model 21.

### 3.8 Ethical approval

This research was carried out based on the voluntary perceptions of public HEI employees and department heads after written letters were sent from each university. Since it is social science research, it is free from experiments that cannot demand ethical approval.

## 4 Results

### 4.1 Preliminary analysis

We investigated and reported the distributional properties of the data for the upper level and lower level using means, standard deviations, skewness and kurtosis among the studied variables (MR-HPWS, procedural justice, PS-HPWS, and engagement) as well as controlling variables. According to Table 2, the skewness and kurtosis tests show that the data are normally distributed. A skewness index greater than 2 and a kurtosis index greater than 7 were the criteria for normal data [147]. Outlier identification was accomplished by assessing the standard deviation less than the mean without outliers (see Table 2).

**Table 2:** Shows the descriptive Statistics of employees.

	Mean	Std. Deviation	Variance	Skewness		Kurtosis	
	Statistic	Statistic	Statistic	Statistic	Std. Error	Statistic	Std. Error
Engagement	3.556	0.801	0.642	−0.1580	0.129	6.99e-4	0.256
Managerial-rated HPWS	3.620	0.532	0.283	−0.2465	0.129	0.226	0.256
Procedural justice	2.928	0.954	0.910	0.2544	0.129	−0.500	0.256
PS-HPWS	3.506	0.591	0.349	0.0406	0.129	−0.226	0.256
Gender	**0.250**		0.389	**8.0919**	0.129	**107.436**	0.256
Age	2.303	0.750	0.563	1.8286	0.129	3.874	0.256
Marriage	1.742		0.270	−0.2322	0.129	−0.365	0.256
Education	3.042		0.235	0.8463	0.129	4.071	0.256
Work_expreince	2.775	0.900	0.810	0.7123	0.129	−0.164	0.256
Salary	5.022	0.453	0.206	−0.9890	0.129	6.936	0.256

### 4.2 Correlation test

According to Table 3, there is no multicollinearity because the correlation values of all the values are less than 0.85 [147]. Again, as shown in Table 3, the Pearson correlations show the expected direction of association, and all the correlations are significant at the P < 0.001, p < 0.01, and p < 0.05 levels. MR-HRM is positively and significantly related to engagement (r = 0.211, p < 0.001) and to PS-HPWS (r = 0.223, p < 0.001), while PS-HPWS is positively correlated with engagement (r = 0.409^,^ p < 0.001). This finding implies that PS-HPWSs mediate the relationship between MR-HPWSs and engagement. Procedural justice is positively related to PS-HPWS (r = 0.668, p < 0.001) and engagement (r = 0.156, p < 0.01), which highlights the moderating effect of procedural justice on PS-HPWS between MR-HPWS and engagement. Therefore, H1 and H2 are expected to have positive relationships.

**Table 3:** Shows Pearson Correlation and Sig. (2-tailed), N-360 employees.

	1	2	3	4	5	6	7	8	9
PS-HPWS	–								
Procedural justice	0.668^c^	–							
Engagement	0.409^c^	0.156^b^	–						
MR-HPWS	0.223^c^	−0.540	0.221^c^	–					
Gender	−0.063	0.015	−0.130	−0.115	–				
Age	0.012	−0.120	0.219^c^	0.180^c^	−0.091	–			
Marriage	−0.139	−0.102	0.085	−0.023	−0.075	0.223^c^	–		
Education	0.050	−0.068	0.230^c^	0.142^b^	−0.099	0.279^c^	0.264^c^	–	
Work_expreince	−0.029	−0.065	0.260^c^	0.075	−0.063	0.679^c^	0.292^c^		–
Salary	−0.081	−0.101	0.098^a^	0.078	−0.138	0.300^c^	0.261^c^	0.449^b^	

Note. Hₐ is positive correlation.

**NB**: coding of Gender: male is coded as “zero” while female was coded “1”

a p < .05.

b p < .01.

c p < .001.

### 4.3 Measurement model

First, after verifying the principal factor analysis items, we applied parceling of items for the three constructs. Orcan [148] classified the advantage of parceling as enhancement of the communality and commonality to uniqueness ratio for each parcel and reduction of the random error, increment of model fit, reduction in the item-specific biases and random error. Accordingly, since scholars have considered exploratory factor analysis (EFA) of HRM second order [14], we developed a second-order HPWS construct through ten human resource management practices categorized under ability-, motivation-, and opportunity-oriented HPWSs. Moreover, engagement is considered a first-order factor, according to previous researchers [14,149]. We also parceled engagement under three categories according to Kahn [142]: cognitive, behavioral and affective (see Table 4). Again, we parceled procedural justice into distributive justice, and Colquit and his colleagues parceled [118] procedural justice [150].

**Table 4:** Shows internal consistency reliability analysis and Explanatory Factor Analysis.

	Items	Cronbach's Alpha	% of variance explained
**HPWS**			
Recruitment and selection (RS)	8	0.808	72.4
Job security (JS)	4	0.762	62.5
Training and development (TD)	8	0.874	81.9
Career ladder and promotion opportunity (CP)	2	0.755	72.3
Performance management (PM)	8	0.834	60.3
Compensation and benefit (CB)	5	0.712	58.9
Participation (P)	7	0.838	85.5
Autonomy (AW)	3	0.722	77.4
Information sharing (ISH)	4	0.825	86.1
Job design (JD)	4	0.768	61.9
**Procedural justice**			
Distributive justice (DJ)	3	0.703	60.9
Procedural justice (PPJ)	6	0.811	54.3
**Engagement**			
Cognitive (CG)	4	0.814	52.7
Affective (AF)	4	0.709	58.1
Behavioral (BH)	4	0.716	62.0

To evaluate measurement, the partial least square structural equation model (PLS-SEM) approach is mostly recommended in social sciences research [151,152]. We employed this method because, in contrast to the factor-based approach, it is a composite-based approach that uses total variance and represents the construct as a linear combination of its indicators [153,154]. Therefore, we employed PLS-SEM. In evaluating the measurement model, researchers [154] have described the following steps.

First, according to Table 5, we verified that factor loadings were acceptable for a newly developed item when it exceeded 0.5, whereas for an established item, the factor loading should be 0.6 or higher [155].

**Table 5:** Show measurement and construct validity.

First order	Second order	Items	Factor loading	AVE	CR	Significance
Ability (A)		RS	0.51	0.59	0.64	**Significant**
		TD	0.96			
Motivation (M)		JS	0.83			
		CP	0.72			
		PM	0.95			
		CB	0.55	0.60	0.66	**Significant**
Opportunity (O)		P	0.81			
		AW	0.84			
		ISH	0.71			
		JD	0.59	0.55	0.62	**Significant**
	PS-HPWS	A	0.81			
		M	,78			
		O	0.87	0.67	0.72	**Significant**
Procedural justice		DJ	0.73			
		PPJ	0.89	0.66	0.71	**Significant**
Engagement		CG	0.74			
		AF	0.76			
		BH	0.85	0.62	0.67	**Significant**

Second, we verified the internal consistency of the constructs using Cronbach's alpha and composite reliability (CR) above 0.70 for both measures [156]. However, a CR of 0.6 is also acceptable for exploratory research [157] (see Table 5).

Third, according to Hair [154], we obtain an AVE greater than 0.500, which implies the extent to which items on a specific construct correlate positively and share a high degree of variance (Table 5).

Fourth, according to Table 6, the correlations are not greater than 0.9 [158], which shows the extent to which our measurements explain the domain of the content [159].

**Table 6:** Shows correlation between constructs.

**Variable 1**	**Variable 2**	**β**	<0.9
Procedural Justice	Engagement	0.169	**Significant**
Procedural Justice	PS-HPWS	0.818	**Significant**
Engagement	PS-HPWS	0.416	**Significant**

Fifth, according to Table 7, the discriminant validity results of Fornell-Larcker show that the square roots of the AVEs on the diagonals are greater than the correlations between constructs [160,161], which shows that the constructs are strongly related to their respective indicators compared to other model constructs [162]. In other words, the correlation square is less than the AVE value. Additionally, the correlations among the constructs are less than 0.90, which indicates that discriminant validity is maintained [156].

**Table 7:** Shows discriminant validity.

No.	Variables	AVE	SQRT of AVE	CR	
	PS-HPWS	0.67	0.82	0.72	**Significant**
	Procedural justice	0.66	0.81	0.71	**Significant**
	Engagement	0.62	0.79	0.67	**Significant**

Sixth, to establish convergent validity, Fornell and Larcker [162] recommended that all factor loadings be significant and should exceed 0.5 for all individual items [163]; the CR for a given factor must exceed 0.6 [157]; and the AVE of each of the constructs must be above 0.50 [156,161]. Additionally, the CR of each construct is greater than the AVE of the respective constructs [163]. Table 7 shows that all preconditions for convergent validity are satisfied.

### 4.4 Common method bias

Since the dependent variable, mediating variable, and moderating variable were measured by one source, this can cause measurement error. Therefore, we assessed and controlled common method bias using procedural and statistical strategies [164].

First, based on the recommendation of Kock et al. [164], we “implement procedural controls in the very first stages of the questionnaire design because employing statistical solutions only afterwards may not be sufficient”. All respondents had more than 2 years of experience; thus, they had sufficient cognitive ability. The interpretability of the questions was checked at the questionnaire development and piloting stage; accordingly, two questions about the engagement variable were adjusted. Again, we employed different respondents for the managerial-rated HPWS (independent) and engagement (dependent) variable measurements; if not, they were the main source of measurement-related common method bias. We also proposed that all the responses be kept anonymous. As much as possible, we tried to keep the surveys as concise as possible in eliminating the idleness of the items. Additionally, we applied items with polar opposites instead of reverse-worded items [165]. Finally, we also applied psychological separation with the aims of masking the researchers’ interest in the independent (PS-HPWS) and dependent (engagement) variables and concealing the relationship between the two [166].

Second, three statistical tests were carried out. First, we tested Harman's single factor test with the rule thumb of less than 50 % of the variance showing no concern of common method bias [164,167]. For engagements, this percentage is 39.177 %; for procedural justice, it is 23.322 %; and for PS-HPWS, it is 47.840, for which the total variance explained is less than 50 %. Second, Tehseen et al. [168] argued that the correlation matrix procedure, as a method of assessing the impact of common method bias through correlation greater than nine of latent variables among principal constructs, implies that there is no convergent validity (see Table 2). Third, to determine how the model fit our data following the recommendation of Hu and Bentler [169]^,^ we calculated multiple indices of fit [170]. A cutoff of x2/df values less than 2.5, Tucker–Lewis index (TLI) and comparative fit index (CFI) values greater than 0.9 [171], and root mean square error of approximation (RMSEA) and standardized root mean square residual (SRMR) values less than 0.08 indicate good model fit [172]. Therefore, the final model fit for the three-factor model (PS-HPWS, procedural justice and engagement) shows the best fit for all alternatives, i.e., 2.195 for the x2/df, 0.951 for the CFI, 0.941 for the TLI, 0.078 for the RMSEA, and 0.079 for the SRMR (see Table 8).

**Table 8:** Shows a result of CFA.

Model	x2/df	CFI	TLI	RMSEA	SRMR
Three-factors model (PS-HPWS, procedural justice, and engagement)	2.195	0.951	0.941	0.078	0.079
Two-factors model (PS-HPWS and procedural justice)	3.651	0.939	0.928	0.086	0.086
Two-factors model (PS-HPWS and engagement)	5.426	0.898	0.880	0.111	0.109
Two-factors model (procedural justice and engagement)	5.644	0.892	0.872	0.114	0.112
One-factor model	5.696	0.892	0.873	0.114	0.111

### 4.5 Structural model

Researchers have employed a multilevel moderated mediation approach to analyze structural models. Procedural justice between departmental levels is considered to moderate the meditational effect of PS-HPWSs between MR-HPWSs and employee engagement using an index of moderated mediation that is a test of linear moderated mediation in path analysis [122].

Preliminarily, when we assess direct effects based on Table 9, there is a positive and significant direct effect of MR-HPW on PS-HPWS [effect or *β=.*499 at <0.001, 95 % CI (0.418, 0.579), SE = 0.0407]. Moreover, the effect of PS-HPWS on the engagement of employees was significant and positive at the department level [effect = 0.614, 95 % CI (0.349, 0.878), SE = 0.133] and within-departments level [effect = 0.371, 95 % CI (0.235, 0.508), SE = 0.069]. To test these direct effects, we applied the centered mean and covariation, which involves partitioning conflated effects into purely between-level and purely within-level effects [173,174] of PS-HPWS and its effect on engagement. However, these are not our main focus.

**Table 9:** Direct effect and moderation effect.

	Effect	SE	p value	LL	UL
rHPWS - > pHPWS	0.4987	0.0407	0.0000	0.4181	0.5792
PJ - > pHPWS	0.1963	0.0448	0.0000	0.1078	0.2849
PJ x rHPWS - > pHPWS	0.1433	0.0126	0.0000	0.1184	0.1683
rHPWS - > ENGM	0.2177	0.1168	0**.0654**	−0.0141	0.4495
pHPWS - > ENGM (B)	0.6135	0.1333	0.0000	0.3492	0.8779
pHPWS - > ENGM (W)	0.3714	0.0692	0.0000	0.2351	0.5077

Note: rHPWS-managerial-rated HPWS, pHPWS-perceived HPWS, PJ-procedural justice, ENGM-engagement, B-between departments, W-within department.

Our first objective, as shown in Table 10, is that the indirect effect of MR-HPWSs on the engagement of employees via PS-HPWSs at the departmental level is significant and positive [indirect effect = 0.306, 95 % CI (0.173, 0.449), SE = 0.071], confirming hypothesis 1. This is analyzed according to multilevel mediation analysis, which proposes that mediation can occur only at the upper level with the help of level 2 variables or MR-HPWSs and aggregation of PS-HPWSs and engagement [173,174]. However, there is no direct effect of MR-HPWSs on engagement, which implies full mediation of PS-HPWSs between MR-HPWSs and engagement.

**Table 10:** Indirect effect and index of moderated mediation.

		Effect	SE	MCLL	MCUL	Significance
H1	rHPWS - > pHPWS - > ENGM (B)	0.306	0.071	0.173	0.449	**Significant**
H2	PJ x rHPWS - > pHPWS - > ENGM (B)	0.088		0.049	0.130	**Significant**

Note: Indirect Effect is Conditional on a Moderator Value of PJ = 0.0000.

Note: rHPWS-managerial-rated HPWS, pHPWS-perceived HPWS, PJ-procedural justice, ENGM-engagement, between departments.

The second objective was to assess the significance and positive interaction effect of perceived procedural justice between MR-HPWSs and PS-HPWSs [*β* = 0.143, 95 % CI (0.118, 0.168), SE = 0.013], based on Table 9. This is the basis for studying moderated mediation effects. The results in Table 10 show a significant and positive index of moderated mediation in which procedural justice moderated the meditation effect of PS-HPWS between MR-HPWS and engagement [effect = 0.088, 95 % CI (0.049, 0.130)]. Hence, hypothesis 2 was proven (see Table 10). To have such an outcome, we carried out a 2-1-1 mediation (MR-HPWSj-PS-HPWSij-engagementij) design with a Level 1 moderator (procedural_justiceij) using a centered cluster strategy that is performed with a variable measured at Level 1, and the group means of these variables are reintroduced at Level 2 (procedural justice, PS-HPWS and engagement) [123,173].

### 4.6 Discussion

To implement both organizational and employee mutual benefits, organizational HPWS must first affect individual-level attitudes and behaviors [6,[19], [20], [21]], including engagement, which is a source of organizational performance [13,14], or organizational effectiveness, creativity, innovation, employee satisfaction, commitment and well-being [17]. However, how HPWSs impact engagement has not been determined. Many researchers have proposed that employees perceive HPWSs [175,176]. Similarly, researchers have recommended multilevel designs focusing on line managers, and employees’ perceptions of the same HPWS can better impact engagement [[177], [178], [179]]. Hence, many researchers have studied the mediating effect of PS-HPWSs on MR-HPWSs and employee outcomes [31,180], but this area is still in its infancy [35]. Specifically, no study has tested the mediation of PS-HPWSs between MR-HPWSs and engagement, even if there are insignificant studies on related concepts, such as employee empowerment [32] and commitment (with firm-level HPWSs) [58]. Relying on and integrating recent works on the impact of MR-HPWSs on PS-HPWSs [181] and the effect of PS-HPWSs on engagement [102], we tested empirical data collected from 102 department heads and 360 employees and found that PS-HPWSs positively mediate MR-HPWSs and engagement based on signal theory. According to signal theory [65], homologous HPWSs that consider integrated dual aspects constitute a new chapter for the current SHRMP world that could minimize the gap between MR-HPWSs and PS-HPWSs. According to this theory, when a line manager sends a strongly integrated dual HPWS to employees, employees perceive it and react with a corresponding attitude or engagement.

Signal theory suggests that inconsistencies can occur between MR-HPWSs and PS-HPWSs and cascades to engage [61]. Since the existing HR literature shows that the ‘intended-actual-experienced’ linkages of HPWSs have not been studied adequately to indicate the source of gaps between MR-HPWSs and PS-HPWSs [31,32,55,96,178,179], there is limited evidence on the underlying reasons for such deviations [57,180]. Previous researchers have employed manager communication quality [55], demographic similarity with managers [37,182], employees' social status [31], employee-supervisor relationships [112], perceived employee-oriented organizational culture and top managers' benevolent leadership behavior (but in relation to firm HPWS [58], cultural value similarity and employees' cognitive cultural intelligence [57]) as moderators between MR-HPWSs and PS-HPWSs. However, these studies have not employed HPWS strength features as moderating variables, as recommended by recent studies [46,48,74,114], specifically consensus [48]. A few studies have considered HPWS features as moderating variables [48,65,114,[183], [184], [185], [186]]. Hence, we studied justice, which is one of nine meta-features of HPWSs, as a moderator of the meditational effect of PS-HPWSs between MR-HPWSs and engagement. In summary, we found that the inconsistency of the mediating effect of PS-HPWSs on the relationship between MR-HPWSs and employee engagement can be strengthened through a few or nine features of HPWS strength theory in the case of Ethiopian public HEIs.

### 4.7 Contributions

#### 4.7.1 Theoretical contributions

Our study contributes to both substantive and methodological theories and empirical works. First, we studied the mediation of PS-HPWSs between MR-HPWSs and engagement using signal overarching theory [60,61]. While the majority of HRM and OB studies have employed signaling in the preemployment process, few studies have applied signal theory to the meditational effect of PS-HPWSs between MR-HPWSs and PS-HPWSs and their impact on employees’ attitudes [61]. Therefore, this research improves the application of signal theory to study the mediation of PS-HPWSs between MR-HPWSs and engagement.

Second, few studies have investigated information asymmetry between MR-HPWSs and PS-HPWSs and its effect on employees’ attitudes [11,48,60,61,74,78]. Our work adds value by focusing on the gap between MR-HPWSs and PS-HPWSs and their effect on engagement [60,61] through consideration of dual-signal fit aspects because doing so minimizes such inconsistency between them [65]. Hence, this study enhances the application of signal theory with a focus on the fit of signals or HPWSs on both MR-HPWSs and PS-HPWSs, consequently resulting in better employee engagement [60,61,78].

Third, as discussed in the literature above, there is a gap between MR-HPWSs and PS-HPWSs in terms of engagement in signal theory [60,61]. Therefore, a low level of employee engagement is related to the inconsistency between MR-HPWSs and PS-HPWSs [45,113] and can be moderated through the strength of HPWSs [46,48,74]. Similarly, according to HR attribution theory, external environment-oriented dual justice (procedural and distributive) has also been described by many scholars [187], but its inclusion in analogous HPWSs by both managers' and employees' experienced studies has not been clearly investigated. Guest et al. [48] studied the moderation of managers’ agreement, which is one of the features of consensus (of the nine meta-features of HPWS strength), whereas we studied perceived procedural justice, which is another feature of consensus (of the nine meta-features of HPWS strength), as a moderating variable between MR-HPWSs and PS-HPWSs and their impact on engagement. Hence, this research addressed this gap, which is a hot issue in the PS-HPWS research world.

Fourth, this study also implies the integration of the perception of HPWS content as a mediator and the HPWS process as a moderator, which responds to the call for research on the integration of these two theories [48,65]. This is partially similar to the findings of researchers who have proposed that perceived HR strength moderates the relationship between HPWS and employee outcomes [[183], [184], [185], [186]]. This approach is opposite to the previous integration of HPWS content under the umbrella of the theoretical features of HPWS strength [45] and as mediating constructs in an understanding of the relationship between organizational HPWS and employee-level outcomes [93].

Fifth, we applied only upper-level moderated mediation while considering the impact of PS-HPWSs on engagement at both the upper and lower levels. Therefore, this study contributes to implementing multilevel moderated mediation analysis under the tent of multilevel structural equation models in the SHRMP, which is a call of researchers repetitively [41]. Therefore, this study extends the application of 2-1-1 multilevel mediation and how lower-level mediation and moderation variables are aggregated at the upper level, particularly by employing centering with a cluster decomposition-first strategy, as it is better than the other two approaches (uncentered and group mean centered) by providing an unconflated coefficient or decomposing between- and within-level effects [173].

Fifth, this study contributes to the empirical world. Using 360 employee and 120 department head data from three Ethiopian public HEIs, we tested the positive effects of 2-1-1 multilevel mediation and multilevel moderated mediation among MR-HPWSs, PS-HPWSs, engagement, and procedural justice. Hence, this enhances multilevel SHRMP application in developing countries, particularly in Ethiopia.

#### 4.7.2 Practical contributions

Our research also has practical value because the mediating effect of PS-HPWSs is related to both employees' outcomes and organizational outcomes. PS-HPWSs influence both individual and organizational performance [14]. According to Meijerink et al. [62], a growing body of research has shown that PS-HPWSs are positively associated with employee performance, which in turn boosts organizational performance. Therefore, human resource management investment in employees reciprocates with a higher level of PS-HPWS, and engagement affects the performance of an organization [14]. Specifically, managers should consider employees’ perceptions of both commitment- and control-oriented HPWSs to better increase the engagement of employees in their performance role and organizational performance [102,188] because both commitment- and control-oriented HPWSs (e.g., performance-based pay) are also related to positive organizational performance [108]. An excessive focus on either commitment or control is associated with a negative effect on the perception of HPWSs and engagement. Therefore, managers should focus on balancing or fitting signals in their implementation.

To engage employees more in their role, managers should pay due attention to the procedural justice of performance management. Procedural justice enhances perceptions of mutual obligations between employers and employees, as it strengthens idiosyncratic beliefs arising from cognitive appraisals of circumstances [188]. Hence, to enhance the impact of MR-HPWSs on PS-HPWSs, managers should practice procedural justice, and a higher level of PS-HPWS improves the engagement of employees. A lack of procedural justice means conflict, which shows that when organizational managers treat employees fairly, employees engage in cascading fairness to society or in decreasing the level of conflict within society [189]. Therefore, to mitigate conflict within their organizations and at the societal level, managers and administrators of HEIs should also focus on procedural justice. This is very important for developing countries such as Ethiopia, where conflict is bubbling.

### 4.8 Limitations and recommendation

The contribution of this study does not mean that this study is free from shortcomings. Hence, the following limitations are related to our study.

First, we studied the effect of MR-HPWSs on PS-HPWSs and their impact on engagement using signal theory. However, signal theory proposes not only the impact of line managers on employees but also the reverse effect of employees on line managers [60,61], which may have a substantial effect on engagement. Therefore, we recommend that further researchers include such a reverse effect on engagement.

Second, signal theory focuses not only on how the synergistic characteristics inside HPWSs create signals [60,72] by tapping into the characteristics of clarity, observability, frequency, and consistency but also on additional factors that are likely to be important for HR-related communication (such as the characteristics of information senders and receivers and the environmental distortion of messages) [11]. Therefore, future researchers should include line manager and employee characteristics and/or other environmental distortions as moderators between MR-HPWSs and PS-HPWSs in relation to employee engagement. Previous studies proposed the inclusion of moderating factors related to these three communication-oriented variables, such as demographics (e.g., age, gender, education, socioeconomic background), intrapersonal characteristics (e.g., beliefs, values, and personality traits), family dynamics (e.g., parental relationships) [93,109,110], and cultural values (e.g., country-level power distance) [52] between MR-HPWSs and PS-HPWSs and the interactive impact on engagement. Therefore, we recommend including manager communication quality [55], employees' social status [31], employee-supervisor relationships [112], perceived employee-oriented organizational culture and top managers' benevolent leadership behavior (but in relation to firm HPWS [58], cultural value similarity and employees’ cognitive cultural intelligence [57], and other proposed variables as moderator/s as moderator/s between MR-HPWSs and PS-HPWSs and their association with engagement).

Third, to decrease the mediation conflict of PS-HPWSs between MR-HPWSs and engagement, we tested procedural justice, which is only one aspect of nine meta-features of HPWSs. However, other researchers [48,74] have recommended including nine features. Empirically [114], studied the moderation of nine features of HPWS strength on PS-HPWSs between MR-HPWSs and engagement. “Idiosyncratic perception of HPWS predict PS-HPWS at employees level when HPWS strength is weak because ambiguous situation are interpreted based on direct experience; on the other hand, strong situation reduces the reliance on individual experiences of HPWS that makes perception of PS-HPWS climate more homogenous with one another” [114]. Hence, future researchers should include other or nine features of HPWSs as moderators of the mediation of PS-HPWSs between MR-HPWSs and engagement.

Fourth, we studied the mediating effect of PS-HPWSs on the relationship between MR-HPWSs and employee engagement, and the mediating effect was moderated by procedural justice (one aspect of nine meta-features of HPWSs). This means that this study integrated only PS-HPWS content and HPWS strength; however, the perception of human resource management also includes the third aspect called HPWS attribution [35,190]. Hence, future researchers could study why human resource management or the perception of HPWS attribution differs between MR-HPWSs and engagement. For example, while the relationship between PS-HPWS content and HR attribution is moderated by HPWS strength [65], the impact of PS-HPWS content on engagement can be mediated by HR attributions [102]. Hence, we propose that the impact of MR-HPWSs on engagement can be mediated first by PS-HPWSs and then by HR attribution, where the impact of PS-HPWS content on HR attribution is moderated by HPWS strength.

Fifth, limitations are related to the methodology. Our study focused only on moderated mediation at the upper level, that is, using only collective/aggregated perceived procedural justice. However, other researchers have studied the impact of MR-HPWSs on PS-HPWSs and their impact on employee outcomes at both level 1 and level 2. Based on the work of researchers [34] who studied mediation both between firms and within firms, we propose that future researchers better consider multilevel mediation and multilevel moderated mediation partitioning between departments and within departments.

Sixth, as this study was carried out using cross-sectional data of 360 employees and 102 department heads from only 3/47 public universities in Ethiopia, the study lacks the rigor of real causality and generalizability. Therefore, we call for more rigor. Again, further research should be conducted using a longitudinal design to better explain the causal effects. We recommend that researchers employ a large sample size from many more public and private sectors as well as data from other sectors. Moreover, this study was carried out in a developing country context, and to make it more generalizable, future studies should include the context of developed countries.

### 4.9 Conclusion

The mediation of MR-HPWSs and engagement by PS-HPWSs has been proposed [93,177], but there is an inconsistency of evidence due to the deviance between MR-HPWSs and PS-HPWSs [31,46,60]. Hence, researchers have proposed that this gap can be filled by examining the integrated dual fit of signals and using the moderating effect of HPWS strength features [65]. Therefore, complementing signal theory with HPWS strength theory, we tested the meditational effect of perceived integrated dual HPWSs between managerial-rated integrated dual HPWSs and decreased engagement when employees’ perceived procedural justice is lower than when it is higher.

## Funding statement

No one organization funded to this research. The research was carried out personally by authors’ financial contributions.

## Data availability statement

The datasets generated during and/or analyzed for the current study can be publicly available by the author upon reasonable request unless they are kept confidential for privacy based on agreements with participants.

## CRediT authorship contribution statement

**Sheref Gogsido:** Writing – review & editing, Writing – original draft, Visualization, Validation, Supervision, Software, Resources, Project administration, Methodology, Investigation, Funding acquisition, Formal analysis, Data curation, Conceptualization. **Demis Getahun:** Writing – review & editing, Writing – original draft, Visualization, Validation, Supervision, Software, Resources, Project administration, Methodology, Investigation, Funding acquisition, Formal analysis, Data curation, Conceptualization. **Zerihun Alemu:** Writing – review & editing, Writing – original draft, Visualization, Validation, Supervision, Software, Resources, Project administration, Methodology, Investigation, Funding acquisition, Formal analysis, Data curation, Conceptualization.

## Declaration of competing interest

The authors declare that they have no known competing financial interests or personal relationships that could have appeared to influence the work reported in this paper.

## Footnotes

1 HR perception is a general term for all kinds of perceived HRM, whereas PS-HPWS is the human resource management system that consists of human resource management practices as a bundle and impacts engagement.

2 Proposed theoretical model to better understand how the synergic characteristics inside HPWS create signals is signaling theory (Connelly et al., 2011; Ehrnrooth & Björkman, 2012).
